# Predictors of Preoperative Tinnitus in Unilateral Sporadic Vestibular Schwannoma

**DOI:** 10.3389/fneur.2017.00378

**Published:** 2017-08-03

**Authors:** Georgios Naros, Joey Sandritter, Marina Liebsch, Alex Ofori, Ahmed R. Rizk, Giulia Del Moro, Florian Ebner, Marcos Tatagiba

**Affiliations:** ^1^Department of Neurosurgery, Eberhard Karls University, Tuebingen, Germany; ^2^Department of Neurosurgery, University of Padova, Padova, Italy

**Keywords:** tinnitus, vestibular schwannoma, predictors, binary logistic regression, hearing impairment, tumor size

## Abstract

**Objective:**

Nearly two-thirds of patients with vestibular schwannoma (VS) are reporting a significantly impaired quality of life due to tinnitus. VS-associated tinnitus is attributed to an anatomical and physiological damage of the hearing nerve by displacing growth of the tumor. In contrast, the current pathophysiological concept of non-VS tinnitus hypothesizes a maladaptive neuroplasticity of the central nervous system to a (hidden) hearing impairment resulting in a subjective misperception. However, it is unclear whether this concept fits to VS-associated tinnitus. This study aims to determine the clinical predictors of VS-associated tinnitus to ascertain the compatibility of both pathophysiological concepts.

**Methods:**

This retrospective study includes a group of 478 neurosurgical patients with unilateral sporadic VS evaluated preoperatively regarding the occurrence of ipsilateral tinnitus depending on different clinical factors, i.e., age, gender, tumor side, tumor size (T1–T4 according to the Hannover classification), and hearing impairment (Gardner–Robertson classification, GR1–5), using a binary logistic regression.

**Results:**

61.8% of patients complain about a preoperative tinnitus. The binary logistic regression analysis identified male gender [OR 1.90 (1.25–2.75); *p* = 0.002] and hearing impairment GR3 [OR 1.90 (1.08–3.35); *p* = 0.026] and GR4 [OR 8.21 (2.29–29.50); *p* = 0.001] as positive predictors. In contrast, patients with large T4 tumors [OR 0.33 (0.13–0.86); *p* = 0.024] and complete hearing loss GR5 [OR 0.36 (0.15–0.84); *p* = 0.017] were less likely to develop a tinnitus. Yet, 60% of the patients with good clinical hearing (GR1) and 25% of patients with complete hearing loss (GR5) suffered from tinnitus.

**Conclusion:**

These data are good accordance with literature about non-VS tinnitus indicating hearing impairment as main risk factor. In contrast, complete hearing loss appears a negative predictor for tinnitus. For the first time, these findings indicate a non-linear relationship between hearing impairment and tinnitus in unilateral sporadic VS. Our results suggest a similar pathophysiology in VS-associated and non-VS tinnitus.

## Introduction

Tinnitus is the second most frequent symptom in vestibular schwannoma (VS) patients (1). Approximately 65–75% of the VS patients complaint about tinnitus (2, 3), in 10% of the patient it is even the presenting symptom (1). Furthermore, tinnitus is a major factor impairing the VS patients’ quality of life (4–6).

The current pathophysiological concept of non-VS tinnitus (e.g., idiopathic tinnitus, tinnitus after auditory or baric trauma) hypothesizes a maladaptive neuroplasticity on a cochlear, brain stem, and/or cortical level as a consequence of (hidden) hearing impairment. These neuroplastic changes are supposed to cause a neuronal hyperexcitability for the residual auditory input resulting in the subjective misperception (7–10). Hence, hearing impairment has been shown to be the strongest predictor for non-VS tinnitus (7–11). In contrast, the pathophysiology of VS-associated tinnitus remains unclear. There are several potential mechanisms of tinnitus generation suggested in the literature (12): (i) ephaptic coupling of cochlear nerve fibers by compression (13), (ii) cochlear dysfunction by ischemia or by biochemical degradation (14), (iii) efferent system dysfunction following compression of the efferent fibers in the inferior vestibular nerve (15), and (iv) cortical reorganization following hearing loss (9). Most studies trying to understand the pathophysiology of VS-associated tinnitus by evaluating its clinical predictors have shown an inverse relationship between tumor size and preoperative tinnitus (3, 12, 16–19). In contrast to non-VS tinnitus, only few studies have shown an association between preoperative hearing impairment and tinnitus (3, 12, 17). In a series of 1,000 patients, the rate of tinnitus was higher in hearing than in deaf patients (74 and 46%, respectively) (3). Another study indicates patients with tinnitus to have better hearing than patients without tinnitus, although not reaching statistical significance (17). Similar data are provided by a study in 941 VS patients showing that patients without hearing loss are less likely to experience tinnitus (12). However, no study was able to prove a correlation between tinnitus and hearing impairment, as measured by audiometric thresholds. In contrast, a correlation between patient’s age and VS-associated tinnitus was observed (12, 17). Finally, several other studies could not establish any predictors of VS-associated tinnitus (4, 5, 20).

We hypothesize, that in case VS-associated tinnitus is based on similar pathophysiological principles as non-VS tinnitus, a distinct relationship between tinnitus and hearing impairment is expected. Reviewing literature several possible explanations for the inconclusive evidence have been identified. Most studies have applied bivariate statistics that might have obscured the factual relationship between hearing impairment and VS-associated tinnitus. First, there is a known interaction between preoperative hearing impairment and VS size (21). Second, a bivariate analysis assumes a linear correlation; however, the available data imply a more complex relationship between tinnitus, tumor size, and hearing impairment (12). Hence, a multivariate analysis is more appropriate to disentangle clinical predictors of VS-associated tinnitus. However, multivariate statistics necessitates large patient numbers that were available only for few studies (3, 12, 17, 18).

The objective of this study was to determine clinical predictors (i.e., patient age, gender, tumor size, and hearing impairment) of VS-associated tinnitus in a large neurosurgical patient cohort using a multivariate logistic regression.

## Materials and Methods

### Patients

All patients enrolled in this retrospective cross-sectional study underwent a neurosurgical removal of a unilateral sporadic VS in the Neurosurgical Department of the University of Tuebingen between January 2008 and January 2015. First, we identified all patients undergoing surgical treatment for intracranial neoplasms of the cranial nerves in this time period (*n* = 702) based on their ICD-10 code (ICD-10: D33.3). 21 patients were excluded due to the histological findings other than VS. Second, we excluded all patients with neurofibromatosis II (ICD-10: Q85.0) and bilateral VS (*n* = 61), patients with relapse or postradiation surgery (*n* = 40) and known contralateral hearing loss [Gardner and Robertson (GR) grading >2, *n* = 8] resulting in 572 patients for bivariate analysis (Table 1). However, pure-tone audiograms were missing in 94 patients. Hence, only 478 complete datasets were included to the final multivariate statistical analysis (Table 2). Preoperatively, all patients received a clinical evaluation of VS-associated symptoms, a hearing evaluation by an ear–nose–throat specialist (pure-tone audiogram and speech discrimination) and a magnetic resonance imaging of the brain. Patients’ characteristics are summarized in Table 1. This study was carried out in accordance with the recommendations of the ethics committee of the Eberhardt Karls University Tuebingen for retrospective studies of data collected as part of routine diagnosis and treatment.

**Table 1:** Patient cohort.

	TN−	TN+	
**Age (years)**	38.5% (184/478) 48.4 ± 14.1	61.5% (294/478) 47.8 ± 11.7	*t*_(476)_ = 0.54; *p* = 0.587

**Gender**			
f	63.6% (117/184)	47.6% (140/294)	*X*_(1)_ = 11.61; *p* = 0.001
m	36.4% (67/184)	52.4% (154/294)	

**Tumor side**			
Left	45.1% (83/184)	466% (137/294)	*X*_(1)_ = 0.10; *p* = 0.750
Right	549% (101/184)	53.4% (157/294)	

**Tumor size (Hannover)**			
T1	3.8% (7/184)	6.8% (20/294)	*X*_(3)_ = 10.18; *p* = 0.017
T2	19.6% (36/184)	21.8% (64/294)	
T3	36.4% (67/184)	44.6% (131/294)	
T4	40.2% (74/184)	26.9% (79/294)	

**Hearing loss (Gardner–Robertson)**			
GR1	47.8% (88/184)	43.2% (127/294)	*X*_(4)_ = 28.81; *p* < 0.001
GR2	22.3% (41/184)	25.2% (74/294)	
GR3	14.7% (27/184)	20.7% (61/294)	
GR4	1.6% (3/184)	7.8% (23/294)	
GR5	13.6% (25/184)	3.1% (9/294)	

**Table 2:** Logistic regression predicting likelihood of tinnitus based on tumor size, tumor side, hearing impairment, and gender.

	*B*	SE	Wald	Df	*p*	Odds ratio (OR)	95% Confidence interval for OR	
							Lower	Upper
GR			24.496	4	0.000			
GR2	0.345	0.252	1.877	1	0.171	1.412	0.862	2.312
GR3	0.643	0.289	4.969	1	0.026	1.903	1.081	3.350
GR4	2.105	0.652	10.418	1	0.001	8.211	2.286	29.489
GR5	−1.026	0.431	5.663	1	0.017	0.359	0.154	0.835
SIZE			12.093	3	0.007			
T2	−0.455	0.504	0.815	1	0.367	0.634	0.236	1.704
T3	−0.370	0.483	0.586	1	0.444	0.691	0.268	1.781
T4	−1.113	0.492	5.109	1	0.024	0.329	0.125	.863
AGE	−0.006	0.008	0.613	1	0.434	0.994	0.978	1.010
GENDER	0.618	0.202	9.359	1	0.002	1.854	1.248	2.754
SIDE	−0.074	0.203	0.133	1	0.715	0.929	0.624	1.382
Constant	0.947	0.605	2.448	1	0.118	2.579		

*Tumor size is T2, T3, and T4 compared to T1; hearing impairment is GR2, GR3, GR4, and GR5 compared to T1; and gender is for males compared to females*.

### Clinical Evaluation

All patients underwent a thorough clinical evaluation of VS-associated symptoms (i.e., hearing impairment, tinnitus, dizziness, balance problems, facial palsy, facial dysesthesia, swallowing difficulties, headache, nausea, and vomiting) by a semi-structured interview by experienced neurosurgeons. Finally, the presence of ipsilateral tinnitus symptoms was dichotomized for statistical analysis (0: no tinnitus, TN−; 1: tinnitus present, TN+).

### Grading of the Hearing Loss

Hearing impairment was classified according to the GR scale (22). As most of the hearing tests were performed outside of our center, details of the used speech discrimination tests were missing (i.e., word lists, masking, etc.). Hence, grading was based exclusively on the results of the pure-tone audiometry (PTA) resulting in five classes: GR 1 (good, PTA 0–30 dB), GR 2 (serviceable, PTA 31–50 dB), GR 3 (non-serviceable, PTA 51–90 dB), GR 4 (poor, PTA 51–90 dB), and GR 5 (deaf, PTA 0 dB). To show the relationship between the absolute hearing impairment and the occurrence of tinnitus, we have reanalyzed the data with narrow binning of the PTA: (1) 0–10 dB, (2) 11–20 dB, (3) 21–30 dB, (4) 31–40 dB, (5) 41–50 dB, (6) 51–60 dB, (7) 61–70 dB, (8) 71–80 dB, (9) 81–90 dB, (10) 91–100 dB, and (11) >100 dB (23).

### Tumor Size Classification

In all patients, a preoperative magnetic resonance image of brain with gadolinium contrast was available, and tumor extent was graded according to Hannover classification (3). VS were classified into four classes: T1 (purely intrameatal), T2 (intra- and extrameatal), T3 (filling the cerebellopontine cistern), and T4 (compressing the brain stem).

### Statistics

All statistical tests were performed using SPSS (IBM SPSS Statistics for Windows, Version 22.0. Armonk, NY, USA: IBM Corp.). Group differences in distribution of clinical attributes such as gender, age, tumor side, tumor size, and preoperative hearing impairment were evaluated by Student’s *t*-test or chi-square test. Binary logistic regression analysis was used to determine the predictive value of gender (GENDER), age (AGE), tumor side (SIDE), tumor size (SIZE), and preoperative hearing impairment (GR) for the occurrence of tinnitus (0: TN+ and 1: TN−). Predictive values of the included variable are provided by their odds ratios (OR) together with the 95% confidence interval (95% CI). Data are shown as mean ± standard deviation (SD). Statistical significance was considered with *p* < 0.05 for each statistical test.

## Results

**Figure 1:**
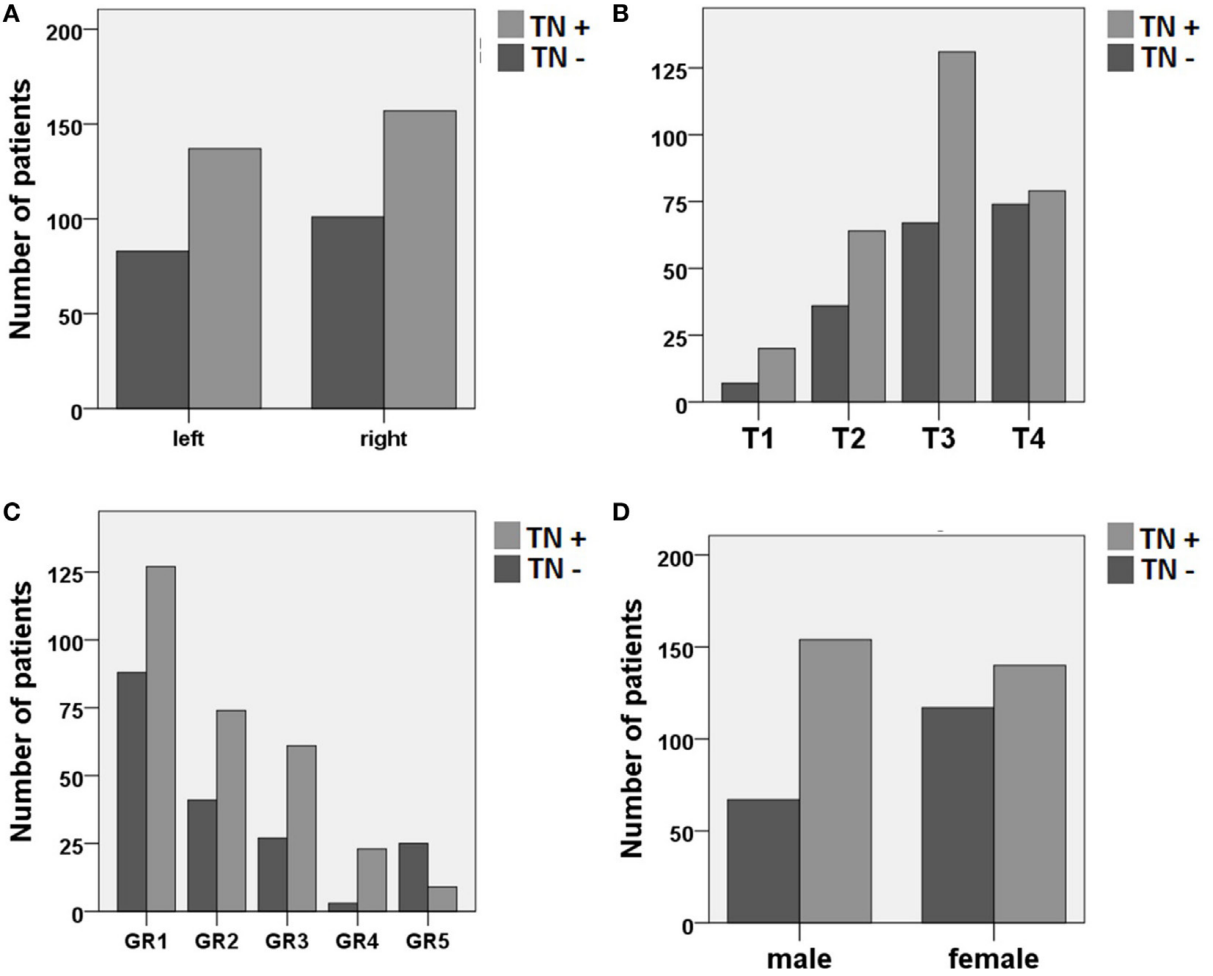
Distribution of patients with (TN+) and without (TN−) preoperative vestibular schwannoma-associated tinnitus depending on **(A)** tumor side, **(B)** tumor size (Hannover classification, T1–T4), **(C)** preoperative hearing impairment (Gardner and Robertson scale, GR1–GR5), and **(D)** gender.

The bivariate analysis showed significant differences between TN+ and TN− patients for gender, tumor size, and hearing impairment. There was a preponderance of male gender, larger tumors (in particular T3), and better hearing function (GR1–GR4) in the TM+ group (Figure 1). There were no significant group differences for age and tumor side (Table 1). To ascertain the effects of gender (GENDER), age (AGE), tumor side (SIDE), tumor size (SIZE), and preoperative hearing impairment (GR) on the likelihood of preoperative VS-associated tinnitus, a binomial logistic regression was performed. The logistic regression model was statistically significant [χ^2^(10) = 52.50, *p* < 0.0001]. Of the five predictor variables GR, SIZE, and GENDER were statistically significant (Table 2). The binary logistic regression analysis identified male gender (*p* = 0.002) and hearing impairment GR3 (*p* = 0.026) and GR4 (*p* = 0.001) as positive predictors. Patients with male gender [OR 1.90 (1.25–2.75)] and higher hearing impairment showed higher odds to exhibit a preoperative tinnitus [GR3: OR 1.90 (1.08–3.35) and GR4: OR 8.21 (2.29–29.50)] than patients with no preoperative hearing impairment (GR1), with severe hearing impairment (GR4) being the strongest predictor of tinnitus. In contrast, large T4 tumors (*p* = 0.024) and complete hearing loss GR5 (*p* = 0.017) were identified as negative predictors. Patients with large T4 tumors [OR 0.33 (0.13–0.86)] and complete hearing loss GR5 [OR 0.36 (0.15–0.84)] were less likely to develop a tinnitus. However, 43.2% (127/294) of the patients with good clinical hearing suffer from tinnitus. In contrast, 26.5% (9/34) of patients with complete hearing loss suffered from tinnitus.

**Figure 2:**
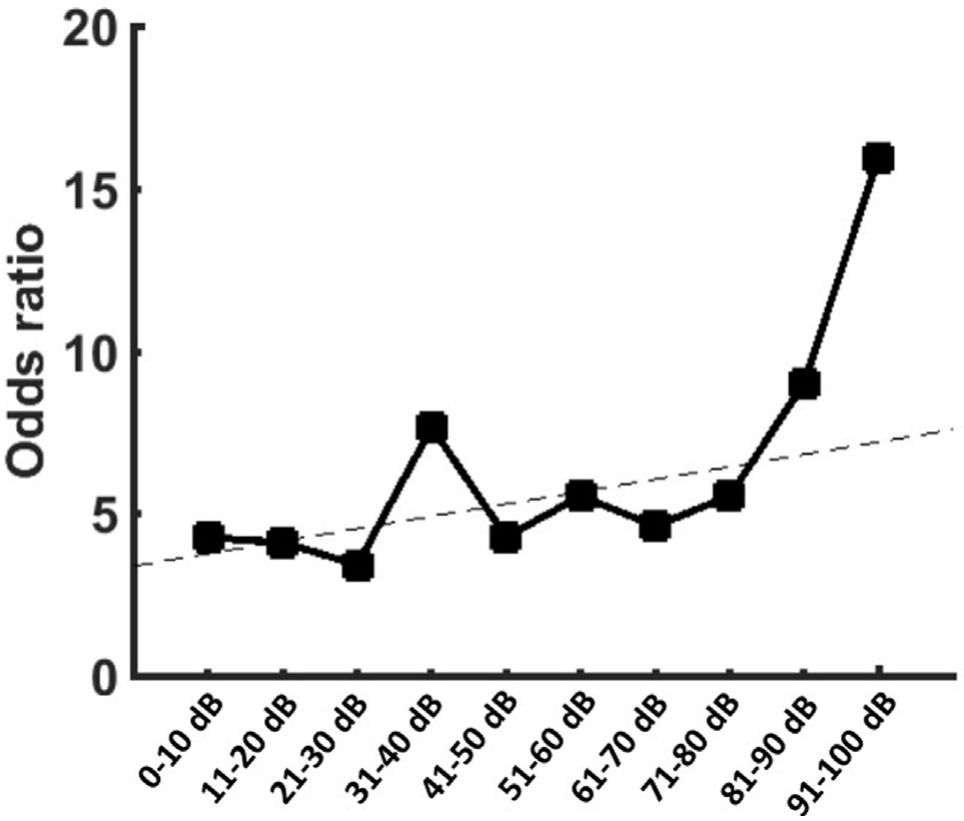
Graph is showing the odds ratios (ORs) provided by the logistic regression for the occurrence of tinnitus when compared to patients with pure-tone audiometry (PTA) >100 dB. Patients with PTA >100 dB showed the smallest risk for tinnitus. In patients with residual function, however, there was an increase of the ORs with increasing hearing impairment.

To proof the increased risk of developing tinnitus depending on the hearing impairment, we reanalyzed the data with narrow binning of the PTA values. A binomial logistic regression was performed to show the predictive value of the PTA for the presence of tinnitus. The logistic regression model was statistically significant [χ^2^(10) = 32.17, *p* < 0.0001] with PTA being a significant predictor of tinnitus (*p* = 0.002). Patients with PTA >100 dB were less likely to present a preoperative tinnitus than other patients (Table 3). However, for patients with residual hearing, there was positive relationship between hearing impairment and the risk of tinnitus occurrence (Figure 2).

**Table 3:** Logistic regression predicting likelihood of tinnitus based on pure-tone audiometry (PTA).

	*B*	SE	Wald	Df	*p*	Odds ratio (OR)	95% Confidence interval for OR	
							Lower	Upper
PTA			27.438	10	0.002			
0–10 dB	1.452	0.527	7.587	1	0.006	4.274	1.520	12.012
11–20 dB	1.419	0.440	10.373	1	0.001	4.131	1.742	9.794
21–30 dB	1.233	0.452	7.440	1	0.006	3.431	1.415	8.322
31–40 dB	2.039	0.481	17.974	1	0.000	7.680	2.993	19.708
41–50 dB	1.457	0.475	9.394	1	0.002	4.293	1.691	10.899
51–60 dB	1.715	0.564	9.253	1	0.002	5.556	1.840	16.771
61–70 dB	1.532	0.533	8.256	1	0.004	4.630	1.628	13.168
71–80 dB	1.715	0.672	6.518	1	0.011	5.556	1.489	20.722
81–90 dB	2.200	0.691	10.128	1	0.001	9.028	2.328	35.003
91–100 dB	2.771	0.667	17.269	1	0.000	15.972	4.323	59.010
Constant	−1.022	0.389	6.907	1	0.009	0.360		

*ORs are provided compared to PTA >100 dB*.

## Discussion

This study evaluated the clinical predictors of preoperative tinnitus in unilateral sporadic VS patients indicating male gender and hearing impairment as positive predictors of tinnitus. In contrast, large tumors and complete hearing loss were disentangled as negative predictors in our patient cohort.

### Relationship between VS-Associated Tinnitus and Tumor Size

So far, most studies evaluating the clinical predictors of VS-associated tinnitus have shown an inverse relationship between tumor size and tinnitus (3, 12, 16–19). In line, our data show no predictive value of tumor size except the fact that patients with extreme large tumors (i.e., Hannover classification T4) are less likely to experience a tinnitus. Most of the available studies include VS patients treated mainly by the so-called translabyrinthine approach covering rather smaller VS (5, 12, 16, 20, 24) In contrast, due to the retrosigmoidal approach used in our department, this study is covering a high proportion of extreme large tumors (i.e., Hannover classification T4) (3, 4, 17, 24, 25). This fact might have contributed to our findings on the relation between tumor size and tinnitus. A detailed look at the statistics indicates that patients with small tumors (i.e., Hannover classification T1) have the largest risk of preoperative tinnitus in comparison to T2–T4 tumors, however, reaching statistical significance only in contrast to T4 tumors. To our opinion, the most likely explanation for this finding is that tinnitus might lead these patients to an early consultation with physicians, resulting in earlier detection of small tumors (3, 17). On the other, as T4 tumors are reaching the brain stem an interaction between tumor and the cochlear nucleus by compression cannot be excluded.

### Impact of Age, Tumor Side, and Gender on VS-Associated Tinnitus

In this study, no correlation between patient’s age or tumor side and VS-associated tinnitus was observed. In contrast, Kameda and colleagues found that patients suffering from VS-associated tinnitus are younger than patients without VS-associated tinnitus (17). Contradictory, Baguley and colleagues have shown a positive correlation between age and tinnitus severity, i.e., greater age being associated with greater tinnitus severity (12). Notably, in our analysis, male gender seems to be a positive predictor of VS-associated tinnitus. While there are some studies claiming a male preponderance in non-VS tinnitus (26, 27), most studies indicate female patients to be more affected (28–30). These gender differences could be related to different stress coping or anxiety sensitivity (30, 31). However, it remains unclear, how these findings refer to VS-associated tinnitus, when the tinnitus is superimposed by a tumor diagnosis and the need to surgical treatment. In such situation, men might exhaust their personal coping resources faster than women. Nevertheless, the relation between tinnitus and gender is still ambiguous. However, most studies demonstrate no association between tinnitus, gender, age, or VS tumor side (4, 5, 18, 20).

### Relationship between VS-Associated Tinnitus and Hearing Impairment

Applying a multivariate analysis, this study is the first one to establish a significant, however, non-linear relationship between hearing impairment and VS-associated tinnitus. Our data show that higher preoperative hearing impairment is increasing the risk of developing a tinnitus while complete deafness, in turn, is reducing the risk. However, our data also show that 60% of the patients with good clinical hearing suffer from tinnitus. On the other hand, approximately 23% of the patients with complete hearing loss are suffering from preoperative tinnitus. For the first time, these findings indicate a non-linear relationship between hearing impairment and VS-associated tinnitus. To our opinion, this finding can be partially attributed to our retrosigmoidal approach, which resulted in a patient cohort with less preoperative hearing impairment (3, 4, 17) than other studies applying mainly the translabyrinthine approach (5, 12, 16, 20, 24). Our results are in good accordance with literature about non-VS tinnitus indicating hearing loss as main risk factor (7–10). Nevertheless, it is well known from non-VS tinnitus that clinical hearing impairment, as measured by the audiometry, is no necessary to develop tinnitus (11, 32, 33). This finding could be explained by the fact that some forms of auditory deafferentation are not discovered by audiometry, e.g., patients with tinnitus who have normal hearing thresholds frequently are shown to have cochlear dead regions or outer hair cell damage compared with controls (10, 32). On the other hand, the sensitivity of the PTA might be insufficient to detect subtle (or hidden) hearing impairment (11, 33). In patients with non-VS tinnitus and normal hearing, Schaette and McAlpine have shown an association between tinnitus and a wave I amplitude reduction of the acoustic-evoked potentials, which is generated by primary auditory nerve fibers. This finding is supposed to be a physiological marker of reduced neural output from the cochlea. At the same time, however, the more centrally generated wave V presented a normal amplitude. These findings were interpreted as a renormalization of neuronal response magnitude to the reduced afferent input within the brainstem compensating the hearing loss (11). Our data show a significant increase of tinnitus occurrence in patients with non-serviceable hearing (GR3 and GR4) indicating that residual, possibly non-functional, activity of the acoustic nerve promotes the occurrence of tinnitus (7–10). In line, there is evidence that cochlear nerve preservation with non-functional hearing seems to predict a new-onset tinnitus after surgical VS removal (18, 34). Finally, although hearing impairment could be the initial source of tinnitus, the subsequent cascade of maladaptive neuroplasticity in the central auditory system is likely perpetuate the tinnitus even without any residual input from the ear. This explains the occurrence of VS-associated tinnitus in patients with complete deafness and the fact that tinnitus is persisting even when afferent input from the ear is eradicated by cutting of the auditory nerve loss (35, 36). In contrast, re-installing functional hearing by cochlear implants has been shown to reduce tinnitus (37, 38).

### Limitations of the Study

A major limitation of the study is the dichotomization of the patients’ tinnitus complaints. Unfortunately, there are no systematic data on the tinnitus severity available loosing important information about the exact relation between tinnitus intensity and hearing impairment. Indeed, there are few studies using dedicated scores in VS-associated tinnitus. For example, Baguley and colleagues have shown a positive correlation between age and tinnitus severity. However, there was no significant association to hearing impairment (12).

## Conclusion

This study proves hearing impairment as the most important predictor of VS-associated tinnitus. This finding suggests similar pathophysiological mechanisms for VS-associated and non-VS tinnitus. A better understanding of the VS-associated tinnitus could pave the way for new therapeutic approaches (e.g., non-invasive neurostimulation).

## Ethics Statement

This study was carried out in accordance with the recommendations of the ethics committee of the Eberhardt Karls University Tuebingen for retrospective studies of data collected as part of routine diagnosis and treatment.

## Author Contributions

GN and FE have contributed to the design of the work, the interpretation of the data, drafting and final approval of the manuscript and have agreed to be accountable for all aspects of the work. JS, ML, AO, AR, and GM have contributed to the acquisition and interpretation of the data, revising and final approval of the manuscript and have agreed to be accountable for all aspects of the work. MT has contributed to the interpretation of the data, revising and final approval of the manuscript and has agreed to be accountable for all aspects of the work.

## Conflict of Interest Statement

There was no external funding of the study. None of the authors have potential conflicts of interest to be disclosed.

## Abbreviations

CI, confidence interval; ENT, ear–nose–throat; GR, Gardner and Robertson; NF, neurofibromatosis; OR, odds ratio; PTA, pure-tone audiometry; VS, vestibular schwannoma.
